# Adsorption of phosphates on a novel eggshell Ca-modified anaerobic sludge-based biochar: Adsorption performance and mechanism

**DOI:** 10.1371/journal.pone.0337183

**Published:** 2025-11-26

**Authors:** Guang-Hui Zhuo, Dong-Xin Xue, Long-Fei Huang, Qi Wang, Guangcan Zhu, Chu-Ya Wang

**Affiliations:** School of Energy and Environment, Southeast University, Nanjing, China; Universiti Teknologi Petronas: Universiti Teknologi PETRONAS, MALAYSIA

## Abstract

Efficient recovery of phosphate from wastewater is required due to phosphorus (P) pollution and resource scarcity. In this study, a Ca-modified biochar (ESBC-1) was developed to enhance phosphate adsorption by co-pyrolysis of digestate from anaerobic fermentation and waste eggshell (1:1 mass ratio) at 700 °C. Under ideal conditions (1 g/L, pH 3–10), ESBC-1’s adsorption capacity fluctuates by less than 8% within this pH range, exhibiting excellent pH adaptability, and the phosphate adsorption capacity of ESBC-1 was 97.74 mg/g, which was 7.9 times higher than that of unmodified biochar (SBC). The effect of common cations (Na ⁺ , K ⁺ , Mg^2^ ⁺ , Ca^2^⁺) and anions (Cl ⁻ , SO₄^2^⁻) on the adsorption was small, but due to competitive binding and pH, CO₃^2^⁻ and HCO₃ ⁻ inhibited the adsorption substantially. The process followed the pseudo-second-order kinetic model (R^2^ = 0.9988), which indicates that chemical adsorption is the rate-limiting step, and Freundlich isotherm model (R^2^ = 0.9763), which reflects heterogeneous adsorption sites on the ESBC-1 surface. At 298 K, the maximum Langmuir adsorption capacity is 529.10 mg/g, while that of SBC is only 287.72 mg/g — this confirms the significant promotion effect of Ca modification. According to characterization (SEM-EDS, FTIR, XRD, XPS), adsorption mostly happened by chemical precipitation, where surface Ca^2^ ⁺ was hydrolyzed from Ca(OH)₂ and combined with phosphate to form insoluble hydroxyapatite (Ca₅(PO₄)₃OH). In the actual biogas (TP = 24.49 mg/L, pH = 8.64), ESBC-1 was used at a dosage of 8 g/L, and phosphate removal rate reached 75.23%. This work demonstrated a sustainable “waste resource utilization-pollution control” strategy by converting sludge and eggshell into an effective phosphate adsorbent, suggesting a new way to address the problems of waste valorization and eutrophication.

## Introduction

The growth of organisms and the healthy functioning of ecosystems depend on phosphorus, which is also crucial for industrial and agricultural processes [1]. Nevertheless, phosphorus emissions and overuse have progressively resulted in a serious phosphorus pollution problem due to the quick development of industry, urbanization, and agriculture [2]. A major environmental issue that is acknowledged worldwide is eutrophication of water bodies, and lakes will become eutrophic when their phosphorus concentration surpasses 0.02 mg/L [3]. Furthermore, phosphorus has finite and unequally distributed reserves and is not a renewable resource [4]. Therefore, it is essential to remove excess phosphorus from water bodies and attempt to recover it in order to lessen the phenomenon of eutrophication of water bodies and to address the issue of phosphorus resource scarcity.

The most common phosphorus removal techniques used today are chemical precipitation, biological processes, and physical techniques. These techniques still need to overcome a number of obstacles, though, including low treatment efficiency, high cost, and possible secondary contamination [5]. Adsorption is regarded as one of the most effective techniques for phosphorus removal in aquatic environments due to its advantages such as low cost, ease of operation, simple design, high removal efficiency, and straightforward adsorbent recovery [6,7]. In recent years, a wide range of innovative and efficient adsorbents—including carbon-based composite materials, modified activated carbon, polymers, and nanomaterials—have demonstrated significant potential in pollutant removal applications [8]. For instance, the development of advanced interfaces in nanocomposites has been shown to greatly enhance adsorption performance [8]. Similarly, biopolymer-based composites, particularly those derived from chitosan and loaded with metals, have emerged as promising adsorbents due to their biocompatibility and high affinity for anions [9]. Particularly, biochar has been used extensively in the field of phosphorus removal from wastewater because of its many sources, ease of preparation, affordability, and effective treatment [10,11]. Biochar is rich in hydroxyl, carboxyl, aliphatic double bonds and aromatic structures [12, 13]. In addition, biochar has a porous structure, which makes it conducive to phosphorus removal from water bodies [14]. Additionally, the finished adsorbed biochar has a lot of potential as a slow-release fertilizer to boost crop growth, enhance soil fertility, and raise agricultural yields [15]. However, because of its negatively charged surface, which restricts its affinity for anions like phosphate, biochar made straight from biomass has a limited capacity to adsorb phosphorus [16]. A study showed that biochar prepared by direct pyrolysis of carrot pomace had little phosphate adsorption capacity [17]. Therefore, research on the modification of biochar has attracted great interest. Biochar modification mainly involves acids [18], bases [19], salts [20] or loaded metals such as Mg [17], Ca [1] and Mg/Al [21]. Numerous studies have shown that biochar modified with metal cations (such as Fe^3+^, Ca^2+^, Al^3+^, Mg^2+^, and La^2+^) has a high affinity for phosphorus in wastewater, often through mechanisms like inner-sphere complexation and precipitation, which are critical for efficient phosphate recovery [22].

According to previous studies, Ca has attracted increasing attention as a metal modifier because of its ecological non-toxicity and abundant properties [23]. Wang et al. modified pristine biochar with Ca(OH)_2_ powder as a Ca source and the results showed that the maximum adsorption of phosphate by the modified biochar could reach 314.22 mg/g [24]. In order to achieve direct Ca^2+^ loading onto the biochar, conventional calcium-modified biochar preparation techniques usually employ pure metal salts like CaCO_3_, Ca(OH)_2_, or CaCl₂ as the calcium source [24–26]. Although the calcium structure of biochar can be controlled with these calcium reagents, their practical application is challenging and expensive. Therefore, it is necessary to find cheap and effective alternatives. This aligns with the principles of clean production and circular economy, which advocate for the valorization of waste streams into valuable resources [27]. In order to implement the concept of using waste to treat pollution, eggshell was used as the calcium source in this study. Eggshell with a CaCO_3_ content of roughly 94% exhibit a lot of promise as a low-cost, Ca-rich material [28]. Furthermore, digestate, a plentiful by-product of anaerobic fermentation, was chosen as the raw material for the production of biochar in this study, which is also consistent with the ideas of waste utilization and economy. However, how to realize eggshell calcium material loading in digestate-based biochar is still a focus in current research. Therefore, this study suggested using co-pyrolysis to load calcium materials from eggshell onto digestate-based biochar.

In this study, Ca-modified biochar was prepared using biogas digestate and eggshells as raw materials and used for the recovery of phosphate. The ideal ratio of eggshell to sludge powder was experimentally established to improve the material preparation conditions. The impacts of biochar dosage, coexisting ions, beginning pH, and real slurry on the adsorption process were further examined, along with the analysis of adsorption isotherms and kinetics. Characterization methods including SEM, SEM-EDS, SBET, FTIR, XRD, and XPS were utilized to elucidate the intrinsic adsorption processes between biochar and phosphates.

### Material preparation and experimental methods

#### Material preparation.

The digestate was dried in an oven at 105 °C to constant weight, and then crushed into powder by the crusher. As modified materials, eggshell was collected from food waste in the cafeteria of Southeast University, cleaned with deionized water, dried, and then crushed into powder by the crusher.

All of the reagents were acquired from Shanghai, China’s Aladdin Industries. By dissolving the equivalent mass of KH₂PO₄ in deionized water and diluting it in a volumetric flask, the phosphate solution with a concentration of 1000 mg/L was created. The phosphate solution at a concentration of 1000 mg/L was diluted to obtain the desired concentration of each phosphate solution. A number of masses of digestate, eggshell powder was weighed separately and the eggshell powder were mixed with the digestate in the eggshell digestate mass ratio of 1:2, 1:1 and 2:1 respectively. The weighed mixture was placed in a tube furnace, which was heated up at 10 °C/min and maintained at 700 °C for 2 h. Ar was introduced throughout to maintain an Ar environment. The surface functional groups of sludge biochar will decrease at a higher temperature, and the CaCO₃ in eggshell is only initially pyrolyzed at 650 °C. Therefore, the pyrolysis temperature was controlled to be 700 °C and maintained for a certain period of time in order to ensure the effectiveness of the modification. The pyrolysis products were washed with deionized water, then dried and milled to obtain pristine biochar (named SBC) and modified biochar from eggshell with the mass ratios of 1:2, 1:1, and 2:1 (named ESBC-0.5, ESBC-1, and ESBC-2).

At the end of the adsorption experiments, the samples were separated by centrifugation, washed three times with deionized water to remove residual ions, and subsequently dried in an oven at 60 °C for 24 h. Samples were collected and named as ESBC-1-P for subsequent characterization.

### Adsorption experiment

The adsorption studies were conducted at a room temperature of 25 ± 2.5 °C unless specified differently. The experiment utilized a biochar dosage of 1 g/L and 30 mL of phosphate solution at a concentration of 100 mg/L for adsorption, with an initial pH of 7. The solution was stirred in an oscillator at 180 rpm for 150 min. Upon completion of the reaction, the adsorbed solution went through filtration via a 0.45 μm membrane, and the phosphate content was measured using the spectrophotometric method.

To investigate the adsorption capacity of different mass ratios of eggshell-modified digestate biochar, 0.03 g of biochar material (ESBC-0.5, ESBC-1, ESBC-2, and SBC) was added to 30 ml of 100 mg/L phosphate solution, respectively. After 150 min of reaction, the phosphate concentration of the filtered solution was measured. The amount of phosphate adsorbed at the moment t (Q_t_, mg/g) was calculated using Eq. (1):


$$Q_{\text{t}}=\frac{V}{M}\left(C_{\text{0}}-C_{t}\right)$$ (1)

Where $Q_{\text{t}}$ represents the amount of pollutant adsorbed at the moment *t*, mg/g; $C_{\text{0}}$ and $C_{\text{t}}$ represent the concentration of pollutant at the initial moment and the moment *t*, respectively, mg/L; V represents the volume of the solution, L; M is the amount of adsorbent dosage, g. When the reaction reached equilibrium, the phosphate concentration in the post-filtration solution was measured and the phosphate adsorption capacity ($Q_{\text{e}}$, mg/g) was calculated using Eq. (2), and the phosphate removal rate (R, %) in the solution was calculated using Eq. (3):


$$Q_{e}=\frac{V}{M}\left(C_{\text{0}}-C_{e}\right)$$ (2)

$$R=\frac{C_{\text{0}}-C_{e}}{C_{\text{0}}}\times 100\%$$ (3)

Where $Q_{\text{e}}$ represents the amount of pollutant adsorbed at equilibrium, mg/g; $C_{\text{e}}$ is the phosphate concentration at equilibrium, mg/L.

In the dosage experiment, the dosage of biochar was controlled to be 0.4, 0.6, 0.8, 1.0, 1.2, and 1.4 g/L, which was added to 100 mg/L phosphate solution. At the end of the reaction, the phosphate concentration of the filtered solution was measured.

In the experiment examining the influence of initial pH on the solution, the pH was set to 3, 4, 5, 6, 7, 8, 9, and 10 using HCl (0.1 M) and NaOH (0.1 M) solutions. Subsequently, biochar was incorporated into each of the 100 mg/L phosphate solutions at the specified pH levels. Upon completion of the process, the final pH of the solution and the phosphate concentration were measured.

The experiment investigating the influence of coexisting ions involved varying concentrations (0.05 mol/L, 0.1 mol/L) of common cations (Na^+^, K^+^, Mg^2+^, and Ca^2+^) and anions (Cl^-^, CO3^2-^, HCO_3_^-^, and SO_4_^2-^) in a mixed solution containing 100 mg/L phosphate, and the adsorption experiments were then conducted at a dosage of 1 g/L of biochar.

In rural areas, the effect of actual biogas slurry on phosphate adsorption by ESBC-1 was also investigated. The specific experimental steps were to determine the total phosphate content and initial pH of the actual biogas slurry. Then, using the gradient addition method (0.33–12 g/L, 9 distinct gradients of addition), and the adsorption capacity of ESBC-1 was examined. The rest of the steps were the same as the former. The ideal dosage for the practical treatment of biogas slurry was ultimately established by computing the equilibrium adsorption capacity and removal rate of ESBC-1.

For adsorption isotherm experiments, 30 ml of phosphate with concentrations ranging from 20 to 1000 mg/L were prepared. The initial pH of the solution was adjusted to 7 with HCl and NaOH prior to conducting the experiments at 298 K. Adsorption isotherm data were obtained using Eq. (4), Langmuir’s equation, and Eq. (5), Freundlich’s equation, in order to obtain adsorption isotherm data:


$$\frac{C_{e}}{Q_{\text{e}}}=\frac{\text{1}}{q_{m}K_{L}}+\frac{C_{e}}{q_{m}}$$ (4)

$$Q_{e}=K_{f}C_{e}^{1/n}$$ (5)

where ${\text{q}}_{\text{m}}$ represents the maximum adsorption capacity (mg/g), and ${\text{K}}_{L}$ is the affinity constant, $K_{\text{f}}$, and 1/n represent constants.

In the adsorption kinetics experiment, 90 mL of phosphate solution with a concentration of 100 mg/L was prepared, to which 0.09 g of biochar was added. Samples were taken at 1, 3, 5, 10, 15, 20, 30, 45, 60, 90, 120, 150 min and phosphate concentrations were measured. Adsorption kinetic data were obtained by fitting the pseudo-primary kinetic equation (Eq. (6)), the pseudo-secondary kinetic equation (Eq. (7)):


$$Q_{t}=Q_{e}\left(1-e^{-k_{\text{1}}t}\right)$$ (6)

$$\frac{t}{Q_{t}}=\frac{\text{1}}{k_{\text{2}}Q_{e}^{\text{2}}}+\frac{t}{Q_{e}}$$ (7)

where ${\text{k}}_{\text{1}}$ and ${\text{k}}_{\text{2}}$ stand for the pseudo-primary and pseudo-secondary kinetic reaction rate constants, respectively, min ⁻ ¹ and g/(mg·min).

### Material characterization and analytical methods

Scanning electron microscopy (SEM) was used to examine the material’s surface morphology (ZEISS Sigma 360, Germany). The materials’ specific surface and pore properties were measured using a fully automated specific surface and porosity analyzer (BET, Micromeritics ASAP 2460, USA). X-ray diffraction (Rigaku SmartLab SE, Japan) was used to examine the material’s microstructure and crystal structure. The functional groups of the materials were examined using Fourier transform infrared spectroscopy (FTIR) in the 4000–400 cm^−1^ wave number range (Thermo Fisher Scientific Nicolet iS20, USA). Using an X-ray photoelectron spectrometer (XPS; Thermo Scientific K-Alpha, USA), the materials’ elemental valence states and surface chemical composition were determined.

## Results and discussion

### Optimization of synthesis conditions

**Fig 1:**
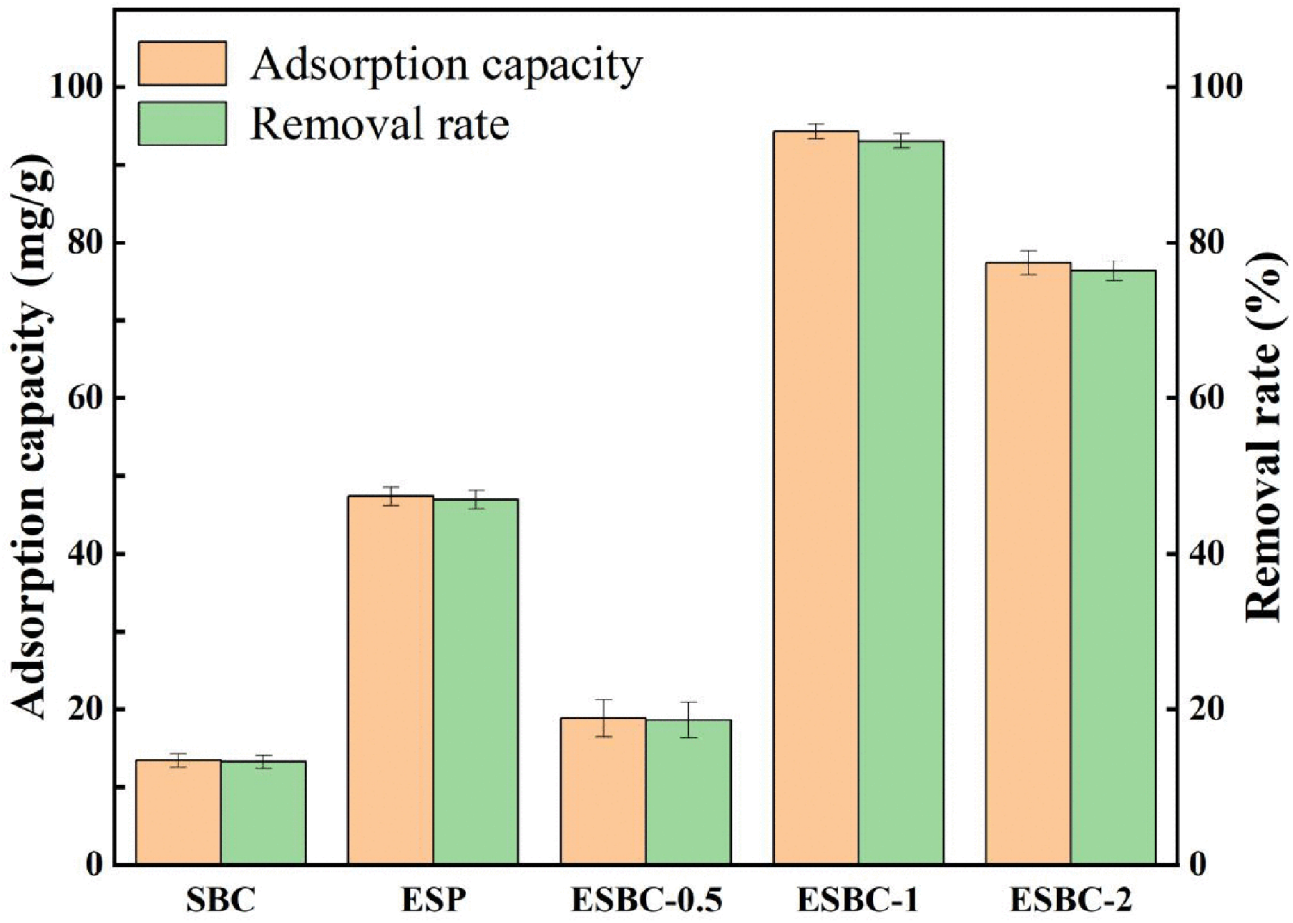
Effect of different mass ratios on the adsorption properties of phosphate (P concentration = 100 mg/L, pH = 7, material dosage = 1 g/L, T = 25 ± 2.5 °C).

The varying mass ratios of eggshell powder and digestate powder will influence the phosphate adsorption efficacy in the synthesis of digestate-based biochar, therefore, optimizing the preparation conditions may enhance phosphate adsorption capacity. Modified biochar was produced by mixing eggshell powder with digestate powder in mass ratios of 1:2, 1:1, and 2:1, followed by calcination to yield samples named ESBC-0.5, ESBC-1, and ESBC-2, respectively. Adsorption experiments with unmodified sludge biochar and pure eggshell powder (ESP) product were also conducted as control groups. The Fig 1 illustrates that the adsorbent changed with varying mass ratios and exhibited distinct adsorption capabilities. The adsorption capacity of the unmodified biochar for phosphate was only 13.42 mg/g with a removal rate of only 13.24%, while the adsorption capacity of the pure eggshell powder product was 47.38 mg/g with a removal rate of 46.98%. Increasing the percentage of eggshell powder substantially enhanced both the adsorption capacity and the removal rate. The adsorption capacity and removal rate were 94.33 mg/g and 93.06%, respectively, at a 1:1 ratio of eggshell powder to digestate powder. Nonetheless, when the ratio was elevated to 2:1, the adsorption capacity and removal rate diminished to 77.40 mg/g and 76.36%, respectively. Obtaining relevant data in Support Information S3 Table. Therefore, the ideal mixing ratio of eggshell powder and digestate powder, calculated by balancing economic feasibility and adsorption performance, was established as 1:1 in the succeeding studies.

### Characterization of sludge-based biochar materials

**Fig 2:**
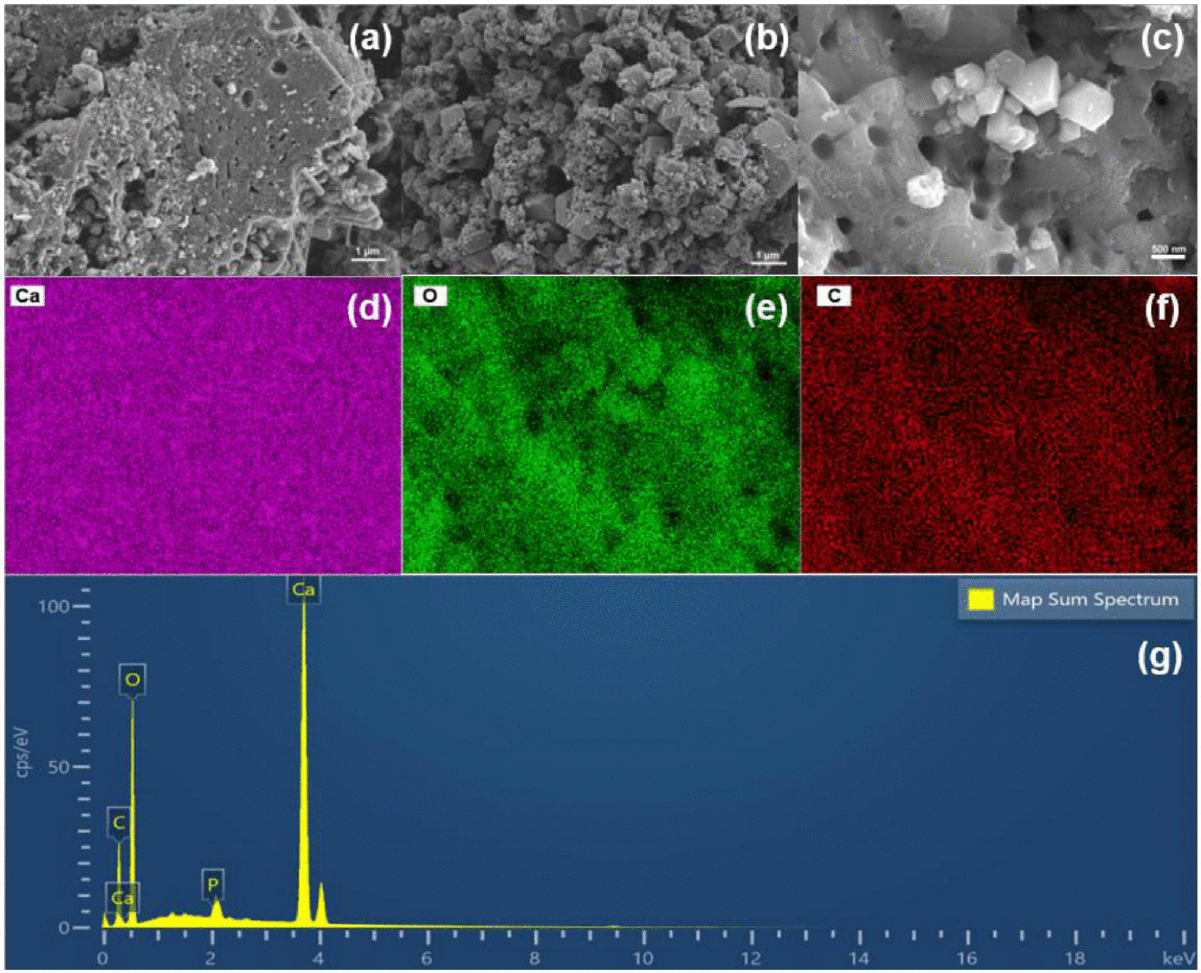
(a) The SEM image of SBC, (b-c) The SEM image of ESBC-1, (d-f) The EDS elemental diagram of ESBC-1 and (i) the Mapping spectrum of ESBC-1.

Fig 2A-2C show the SEM images of SBC and ESBC-1. The pristine SBC’s surface structure is comparatively dense, as shown in Fig 2A. After modification, the surface of ESBC-1 became noticeably rough and granular, as shown in Figs 2B and 2C. For the SEM image of ESBC-1 before adsorption, numerous block-like particles are distributed on the surface, which may be the generated Ca(OH)₂ particles. Ca(OH)₂ nanoparticles are formed by the reaction between CaO and the hydroxyl groups of biochar [29]. The surface particles produced during the modification process exhibit obvious agglomeration characteristics. This particle agglomeration phenomenon caused the surface roughness of the material to increase substantially [30].

Using SEM-EDS analysis, elemental imaging characterization of ESBC-1 was carried out in order to further examine the elemental distribution characteristics (Figs 2D-2G). According to mapping spectrum results, Ca makes up 35.9% of the mass fraction, and C, O, and Ca all showed overlapping spatial distribution characteristics on the material surface. This semi-quantitative analysis by SEM-EDS demonstrated that the co-pyrolysis process can successfully encourage the oriented deposition of Ca-containing minerals on the surface of the biochar matrix, forming structures with distinctive morphologies, when the sludge is thermally modified at 700 °C.

**Fig 3:**
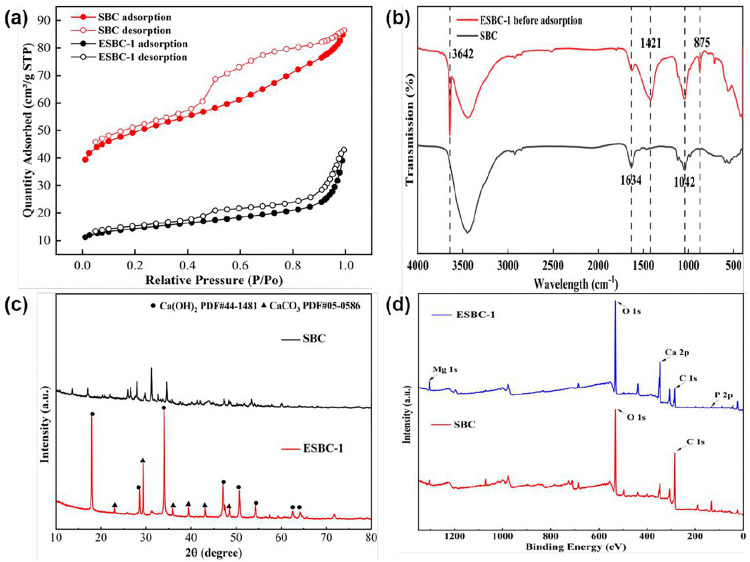
(a) Adsorption and desorption curve, (b-d) FTIR spectra, XRD patterns, and XPS spectra of SBC and ESBC-1.

The parameters of S_BET_, average pore diameter (APD), and total pore volume of SBC, ESBC-1, and ESBC-1-P (after phosphate adsorption) are displayed in Table 1. Fig 3A displays the adsorption-desorption isotherms curve that were acquired for both materials at 77 K. The ESBC-1 material’s surface area and porosity were lower than those of the pristine biochar (SBC). Although SBC’s BET surface area (181.95 m^2^/g) was substantially larger than ESBC’s (52.28 m^2^/g), its adsorption capacity (12.37 mg/g) was substantially lower than ESBC-1’s (97.74 mg/g). It implied that the primary elements influencing the adsorption process are neither physical adsorption nor spatial pore structure [31]. The specific surface area and surface roughness of ESBC-1-P both substantially grew as the adsorption process went on. These modifications were ascribed to the adsorption-induced creation of new phosphate crystals on the surface of the biochar.

**Table 1:** Comparison of S_BET_, Total pore volume, and APD for SBC, ESBC-1, and ESBC-1-P (after phosphate adsorption).

Parameter	Adsorbents		
	SBC	ESBC-1	ESBC-1-P
S_BET_ (m^2^/g)	181.95	52.28	180.54
Total pore volume (cm^3^/g)	0.083	0.052	0.081
APD (nm)	4.50	8.49	4.43

The chemical bond structures of SBC and ESBC-1 were characterized and analyzed using FTIR (Fig 3B). The functional groups of ESBC-1 changed obviously after the addition of eggshell as compared to SBC. The FTIR spectral band near 3642 cm ⁻ ¹ belongs to the stretching vibration of -OH, which is associated with the presence of Ca(OH)_2_ [32]. The enhancement of the -OH peak in the material of the eggshell-modified material compared to the unmodified virgin sludge biochar suggested that the O-H group of Ca(OH)₂ is enhanced due to eggshell modification. A new characteristic peak at 1421 cm ⁻ ¹ appeared after modification, which was derived from the asymmetric stretching vibration of CO_3_^2^ ⁻ , indicating that the eggshell modification successfully introduced CaCO_3_. In addition, the characteristic peak of ESBC-1 at 1042, 875, 713 cm^-^¹ was enhanced compared with that of SBC. The characteristic peak at 1042, cm^-1^ is due to the P-O stretching vibration, and the enhancement of the characteristic peak here for the adsorbed material indicated that the phosphate was successfully adsorbed onto the surface of the material and formed a Ca-P precipitate (e.g., Ca_5_(PO_4_)_3_OH) [33].

The progression of the crystal structures on the surfaces of SBC and ESBC-1 were systematically analyzed using X-ray diffraction (XRD). As shown in Fig 3C, compared to SBC, ESBC-1 show characteristic diffraction peaks at 2θ = 22.98°, 29.34°, which can be attributed to CaCO_3_. The characteristic peak of Ca(OH)_2_ present in SBC was also enhanced to varying degrees after modification. The difference in X-ray patterns between SBC and ESBC-1 suggested that calcium contained in eggshell was successfully introduced into SBC in the form of CaCO_3_ and Ca(OH)_2_. The presence of Ca(OH)_2_ indicated that CaCO_3_ first pyrolyzes to CaO and CO_2_, and then the resulting CaO reacts with moisture and hydroxyl groups associated with the biochar to form Ca(OH)_2_ [29].

The material’s elemental makeup was analyzed using XPS. The energy spectrum study (Fig 3D and Table 2) indicates that ESBC-1 predominantly comprises C (32.55%), O (46.30%), and Ca (16.04%), with trace quantities of Si (1.30%), N (1.73%), and P (2.07%). The Ca concentration of 16.04% suggested the presence of a metal oxide/hydroxide loading structure on the surface, which might substantially improve the material’s ligand adsorption capability for phosphate in water.

**Table 2:** The elemental composition (atomic %) measured by XPS.

Adsorbent	C	O	Ca	Si	N	P
SBC	51.47	31.86	4.13	1.93	3.84	6.77
ESBC-1	32.55	46.30	16.04	1.30	1.73	2.07

### Adsorption isotherm and kinetics

#### Adsorption isotherm.

**Fig 4:**
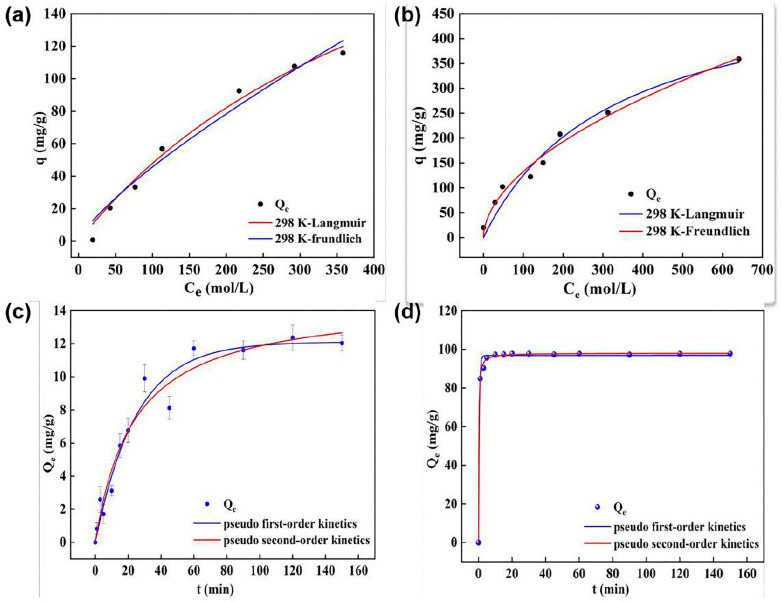
(a-b) Adsorption isotherm models fitting for phosphate adsorption by SBC and ESBC-1, (c) Kinetic models fitting for phosphate adsorption by SBC, (d) Kinetic models fitting for phosphate adsorption by ESBC-1 (P concentration = 100 mg/L, pH = 7, material dosage = 1 g/L, T = 25 ± 2.5 °C).

The isothermal adsorption models systematically elucidated the differences in phosphate adsorption processes between SBC and ESBC-1 (Figs 4A and 4B). The fitting results of the Freundlich and Langmuir models demonstrated that the adsorption behavior of ESBC-1 aligned more closely with the Freundlich model (R^2^ = 0.9763), indicating that the adsorption process happened in a multilayer manner with heterogeneous surface energy distribution and a complex mechanism. The model parameter 1/n=0.5285 (0<1/n<1) further illustrated that the material exhibits optimal site heterogeneity, facilitating the spontaneous adsorption process [34], as shown in Table 3. Conversely, the adsorption characteristics of SBC were more well represented by the Langmuir monolayer adsorption model (R=0.9813), signifying a pronounced homogeneity of its surface-active sites. The alteration in the adsorption process may arise from the development of surface functional group reconfiguration induced by Ca calcination modification. The data for this part of the experiment can be found in the Supporting Information S2 Table.

**Table 3:** Parameters of adsorption isotherm models for phosphate adsorption by ESBC-1 and SBC at 298 K.

Model	Parameter	ESBC-1	SBC
Langmuir	*q*_e_ (mg·g^-1^)	529.10	287.72
	*K*_L_ (L·mg^-1^)	0.0031	0.0020
	*R* ^2^	0.9553	0.9813
Freundlich	*K*_F_	13.79	1.8856
	1/n	0.5285	0.7803
	*R* ^2^	0.9763	0.9657

#### Kinetics.

The adsorption kinetics fitting curves for ESBC-1 and SBC are shown in the Figs 4C and 4D; the adsorption capacity of ESBC-1 for phosphate quickly escalated within 10 min and reached equilibrium thereafter. The adsorption capacity of SBC for phosphate progressively increased, but the adsorption rate started to decrease after 80 min. Pseudo-first-order and pseudo-second-order kinetic models are relevant to the adsorption capacity process of ESBC-1 for phosphate, with the fitting results presented in the Table 4. The adsorption of ESBC-1 on phosphates was more accurately represented by the pseudo-second-order kinetic model than the pseudo-first-order kinetic model. The R^2^ value derived from the simulation employing the pseudo-second-order kinetic model (0.9988) surpasses that from the pseudo-first-order kinetic model (0.9941), signifying that the pseudo-second-order kinetic model more precisely characterizes the adsorption process of ESBC-1 on phosphates. The fitting results demonstrated that phosphate adsorption was primarily governed by intermolecular interactions, electron transfer between the adsorbent and adsorbate, and the synthesis of new compounds, which may interact with phosphate on the surface of ESBC-1 to form new compounds or create electrostatic forces [35]. The R^2^ value of the pseudo-first-order kinetic model for SBC adsorption of phosphate (R^2^ = 0.9601) surpassed that of the pseudo-second-order kinetic model (R^2^ = 0.9569), suggesting that the adsorption capacity of SBC for phosphate is predominantly governed by physical forces. The relevant data can be found in the support information S1 Table.

**Table 4:** ESBC-1 and SBC adsorption kinetic parameters of phosphate.

Model	Parameter	ESBC-1	SBC
Pseudo-first-order	*q*_e_ (mg·g^-1^)	96.83	12.11
	*k*_1_ (min^-1^)	2.07	0.04
	*R* ^2^	0.9941	0.9601
Pseudo-second-order kinetic	*q*_e_ (mg·g^-1^)	98.05	14.64
	*k*_2_ (g·mg^-1^·min^-1^)	0.062	0.0029
	*R* ^2^	0.9988	0.9569

### Experiments on factors affecting phosphate adsorption by ESBC-1

#### Effect of biochar dosage on phosphate adsorption.

**Fig 5:**
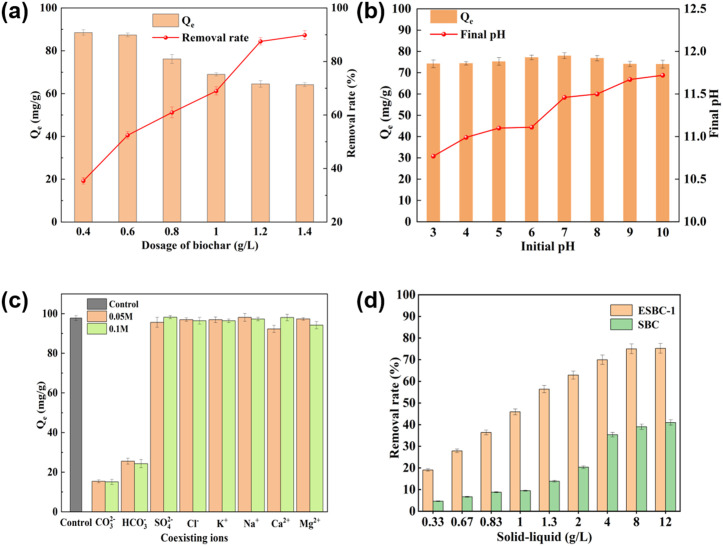
(a) Effect of dosage on phosphate adsorption, (b) Effect of initial pH on phosphate adsorption by ESBC-1, (c) Effect of coexisting ions on phosphate adsorption, (d) Phosphate adsorption performance of ESBC-1 and SBC in real biogas slurry (P concentration = 100 mg/L, initial pH = 7, material dosage = 1 g/L, T = 25 ± 2.5 °C unless specified differently).

The impact of varied dosages on the phosphorus adsorption effectiveness of ESBC-1 was examined by altering the dosage of ESBC-1 utilized in the experiment. The outcomes are depicted in Fig 5A. As the adsorbent dosage was gradually raised from 0.4 g/L to 1.4 g/L, the adsorption capacity exhibited a declining trend, dropping from 88.49 mg/g to 64.17 mg/g. Nonetheless, the phosphate removal rate substantially increased from the initial 35.40% to 89.87% and then stabilized. Concerning the system’s dynamic equilibrium and adsorption mechanism, the adsorption capacity per unit mass of ESBC-1 diminished due to particle aggregation or the underutilization of active sites; however, the total number of adsorption sites in the system increased substantially with higher dosages, thereby enhancing the overall phosphate removal capacity. The removal rate was optimal when the adsorbent dosage reached 1 g/L. At this dosage, the material’s adsorption capacity attained 70.95 mg/g, while its removal rate reached 69.0%. While raising the dosage may enhance the removal rate, an excessive amount of adsorbent indicates a misallocation of resources. Consequently, based on the concepts of adsorption efficiency, adsorption capacity, and cost-effectiveness, 1 g/L was ultimately determined to be the best dosage. The data for this part of the experiment can be found in the supporting information S4 Table.

#### Effect of pH on phosphate adsorption.

The environmental pH greatly impacts the adsorption process. The influence of solution pH on ESBC-1 adsorption of phosphorus was examined and the result is shown in Fig 5B. The adsorption capacity of ESBC-1 for phosphorus increased from 74.2 mg/g at pH 3 to 77.9 mg/g at pH = 7, subsequently reduced to 76.8 mg/g at pH = 8, and further declined to 74.0 mg/g at pH = 10. Therefore, within a wide initial pH range of 3–10, ESBC-1 exhibits good adsorption performance for phosphorus. The pH of the solution can influence phosphorus ionization equilibrium; at different pH values, phosphates exist in different forms, which can affect the adsorption efficiency of the phosphorus adsorbent. Phosphates in aqueous solutions are dominated by H_3_PO_4_ (pH < 2.15), H_2_PO_4_⁻ (2.15 < pH < 7.20), HPO_4_^2^⁻ (7.20 < pH < 12.33), and PO_4_^3^⁻ (pH > 12.33), contingent upon the pH value. [36]. The most easily adsorbed of these three phosphate forms onto the adsorbent surface is PO_4_^3^ ⁻ , which is followed by HPO₄^2^⁻ and H₂PO₄⁻ [37]. The reduced adsorption ability of HPO_4_^2^⁻ and H_2_PO_4_ ⁻ compared to PO_4_^3^ ⁻ could be attributed to the interference of H⁺ in the complexation of Ca with phosphorus [37]. Consequently, elevating the solution pH enhances the binding of Ca to phosphate; however, the reduction in phosphate adsorption capacity when the initial pH surpasses 8.0 may be attributed to the increased concentration of OH^-^ in the solution, which competes with phosphate at the adsorption sites. Moreover, Ca active constituents enhance phosphorus adsorption on ESBC-1 through strong chemical interaction processes, leading to a substantial adsorption capacity of ESBC-1 for phosphorus throughout an extensive pH range (3–11). Similar result was achieved in the research conducted by Deng et al [38], where calcium biochar synthesized from marble waste showed a high adsorption capacity for phosphorus in a wide range of initial pH values from 3 to 11. It is worthy of note that the pH value of the water sample collected at the adsorption equilibrium state is much higher than the initial pH value. This phenomenon may be caused by the exchange of hydroxyl and phosphate ligands, a process that releases CO_3_^2-^ into the solution, causing the pH of the solution to rise. The data for this part of the experiment can be found in the supporting information S5 Table.

#### Effect of coexisting ions on phosphate adsorption.

The adsorption performance of the material will be impacted by a variety of anions and cations present in the water, such as CO_3_² ⁻ , HCO_3_ ⁻ , SO_4_^2^ ⁻ , Cl ⁻ , K ⁺ , Na ⁺ , Ca^2^ ⁺ , Mg^2^ ⁺ , and a variety of other ions. Experiments were conducted to investigate the effect of different ions on phosphate adsorption and the results are shown in the Fig 5C. The results show that the presence of these common ions affects the adsorption properties of the materials to varying degrees. In the anion influence experiment, SO_4_^2^⁻ and Cl ⁻ had no obvious influence on the adsorption effect of the material, which could reach more than 97.8% of the adsorption effect of the control group no matter the concentration of 0.05 M or 0.1 M. However, CO_3_^2^⁻ and HCO_3_ ⁻ had an obvious inhibitory influence on the adsorption effect. When the CO_3_^2^ ⁻ concentration was 0.05 M, the adsorption effect was only 15.7% of the control group, and at 0.1 M, it was 15.4%. The adsorption effect was only 26.1% of the control group when the concentration of HCO_3_ ⁻ was 0.05 M and 24.8% of the control group when the concentration was 0.1 M. In cation influence experiment, the effect of the presence of K^+^ and Na^+^ on the adsorption effect was not obvious, which could reach more than 98.6% of the adsorption effect of the control group at either 0.05 M or 0.1 M concentration conditions. Ca^2+^ had a slight inhibition effect on the adsorption effect under the concentration of 0.05 M, which was 94.4% of that of the control group, and there was no obvious effect under the condition of 0.1 M. Mg^2+^ had a slight inhibition effect on the adsorption effect under the concentration of 0.1 M, which was 96.3% of the control group, and there was no obvious effect under the condition of 0.05 M. The above phenomenon may stem from the following mechanisms: (1) Ligand complexation: the addition of HCO_3_^-^ and CO_3_^2-^markedly reduced phosphate adsorption capacity because they competed for phosphate adsorption sites and combined with Ca^2+^ on the surface of ESBC-1 to form slightly soluble or insoluble substances [37]. (2) CO_3_^2^ ⁻ had the most negative impact on adsorption of all the ions because it caused the pH of the solution to rise sharply, deprotonating the hydroxyl groups on the surface of ESBC-1 and causing electrostatic repulsion with the phosphate anion. The data for this part of the experiment can be found in the supporting information S6 Table.

#### Phosphorus adsorption in actual biogas slurry by ESBC.

In this part of the experiment, the adsorption performance of ESBC-1 on total phosphorus was evaluated using actual biogas slurry. The experiment is conducted with farmyard manure biogas slurry (TP = 24.49 mg/L, pH = 8.64 ± 0.01), and as shown in the Fig 5D, with the dosage of ESBC-1 increased from 0.33 to 12 g/L, the phosphate removal rate was increased from 19.03% to 75.23%. The removal efficiency stabilized at a dosage of 8 g/L. Therefore, 8 g/L was determined to be the optimal economic dosage by analysis. The experimental results for the SBC-addition group were similar to those for the ESBC-1-addition group, with the dosage of SBC increasing from 0.33 to 12 g/L, the phosphate removal rate increased from 4.63% to 40.95%. Similarly, when the SBC dosage reached 8 g/L, the phosphate removal rate tended to stabilize. Phosphorus removal from the actual biogas slurry was reduced compared to the simulated wastewater system. The existence of competing substances, such as metal ions, organic acids, etc., in the biogas slurry’s composite matrix may be the cause of this discrepancy. These chemicals vie with phosphates for the active sites on the surface of the material (Ca(OH)_2_, metal oxides), leading to a decrease in adsorption capacity. Consequently, in the reality of the project, the operational parameters must be optimized based on the water quality characteristics, and the minimum dosage in line with emission standards, such as TP ≤ 0.5 mg/L, should be established through preliminary testing to balance treatment efficacy and operational costs. The data for this part of the experiment can be found in the supporting information S7 Table.

### Studies on the mechanism of phosphate capture

**Fig 6:**
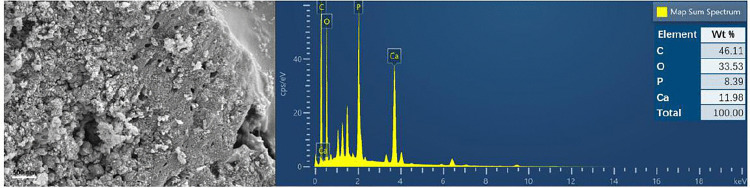
SEM-EDS image of ESBC-1 after phosphate adsorption.

The modification results and adsorption phenomena of the ESBC-1 was analyzed in depth to further speculate on the mechanism of phosphorus adsorption by ESBC-1. The SEM-EDS image of ESBC-1-P (the sample after adsorption) is shown in Fig 6. Compared with the EDS of ESBC-1, the sample after adsorption of phosphate showed a distinct P peak. The increase in P content in ESBC-1-P further confirmed its excellent adsorption capacity. SEM-EDS images of ESBC-1-P show new irregularities on the surface of ESBC-1, which may be due to precipitation between Ca^2+^ and phosphate on the surface. For the adsorbed samples, a large number of flocculent precipitates formed on the surface of the adsorbent. These precipitates aggregated to form a typical Ca_5_(PO_4_)_3_OH (hydroxyapatite) columnar structure, which was subsequently confirmed by other characterization analyses.

**Fig 7:**
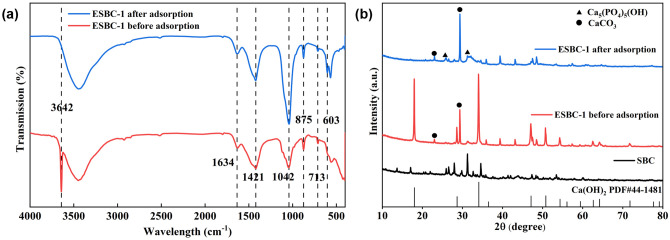
(a) FTIR spectra of ESBC-1 before and after phosphate adsorption, (b) XRD spectra of ESBC-1 before and after phosphate adsorption.

In order to clarify the interfacial chemical behavior of ESBC-1 during phosphate adsorption, ESBC-1, and ESBC-1-P were systematically analyzed using FTIR (Fig 7A). The absorption peak at 3642 cm ⁻ ¹ in the ESBC-1 sample before phosphate adsorption is ascribed to the stretching vibration of -OH, indicating the existence of Ca(OH)_2_. Following the reaction with P, the peak at 3643 cm ⁻ ¹ vanished, signifying that the -OH group has engaged with PO_4_^3^ ⁻ . This demonstrated that Ca(OH)_2_ nanoparticles were the most active sites for phosphate adsorption. Following phosphate adsorption, the peak at 1042 cm ⁻ ¹, indicative of the bending vibration of the P-O group, exhibited a significant rise in intensity, correlating with the production of Ca_5_(PO_4_)_3_(OH) crystals [39]. The distinctive peak of ESBC-1-P at 566 cm ⁻ ¹ corresponds to the bending vibration of the P-O bond and the stretching vibration of the Ca-O-P bond in metal phosphate (Ca-PO_4_), so confirming that phosphate builds stable coordination compounds by chemical adsorption with Ca^2^⁺ [40].

Additional XRD compositional analysis was performed on ESBC-1 and ESBC-1-P, with the findings illustrated in Fig 7B. Before adsorption, the crystallinity of Ca(OH)_2_ and CaCO_3_ in ESBC-1 was notably high. Following adsorption, the strength of the Ca(OH)₂ diffraction peak diminished markedly, and a new diffraction peak, Ca_5_(PO_4_)_3_(OH), emerged, attributable to Ca-P precipitation. This suggested that the liberated Ca^2^ ⁺ interacted with PO_4_^3^⁻ to create Ca_5_(PO_4_)_3_(OH) crystals in the presence of hydroxide ions [29].

**Fig 8:**
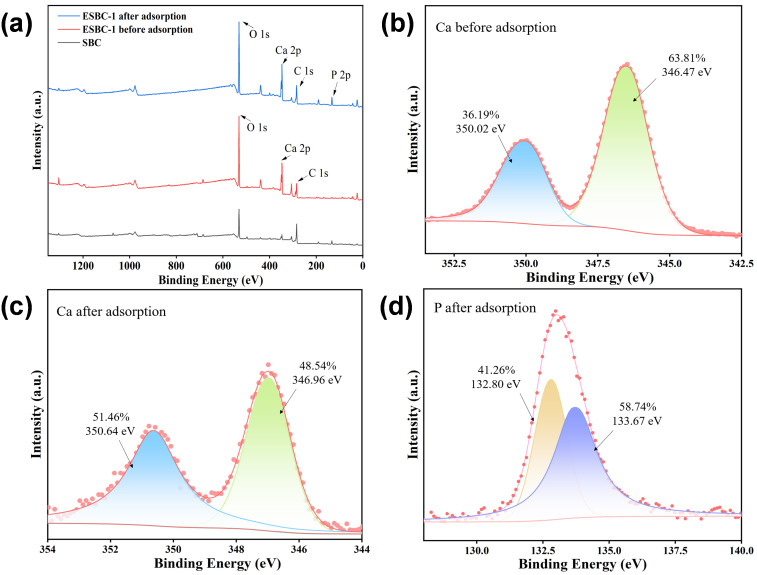
(a) XPS spectra of ESBC-1 before and after phosphate adsorption, (b) Ca 2p spectra of ESBC-1 before phosphate adsorption, (c) Ca 2p spectra of ESBC-1 after phosphate adsorption, (d) P 2p spectra of ESBC-1 after phosphate adsorption.

XPS characterization elucidated the adsorption mechanism of ESBC-1 for phosphate. Following phosphate adsorption, P 2p peaks emerged in the comprehensive XPS spectrum of ESBC-1-P (Fig 8). The Ca 2p of the adsorbed material may be categorized as Ca (2p 1/2) and Ca (2p 3/2) atomic orbitals, associated with peaks at 346.47 eV and 350.02 eV, respectively, indicative of Ca_5_(PO_4_)_3_(OH) (hydroxyapatite) [41].The P 2p spectra can be decomposed into two peaks located at binding energies of 132.80 eV (PO_4_^3-^) and 133.67 eV (H_2_PO_4_^-^). The results demonstrate that chemical precipitation is the main mechanism of phosphate adsorption.

The research indicates that the primary adsorption process of phosphate on ESBC-1 material involves the interaction of Ca^2^⁺ with phosphate, resulting in hydroxyapatite precipitation. The synthesis of Ca_5_(PO_4_)_3_(OH) may be represented by Eq. (8) - (10) [37,42]:


$$5C{\text{a}}^{2+}+3{PO}_{\text{4}}^{3-}+{OH}^{-}\to {Ca}_{\text{5}}{\left({PO}_{\text{4}}\right)}_{\text{3}}OH↓$$ (8)

$$5{Ca}^{2+}+3{HPO}_{\text{4}}^{2-}+4{OH}^{-}\to {Ca}_{\text{5}}{\left({PO}_{\text{4}}\right)}_{\text{3}}OH↓+3H_{\text{2}}O$$ (9)

$$5{Ca}^{2+}+3H_{\text{2}}{PO}_{\text{4}}^{-}+7{OH}^{-}\to {Ca}_{\text{5}}{\left({PO}_{\text{4}}\right)}_{\text{3}}OH↓+6H_{\text{2}}O$$ (10)

## Conclusions

To address the severe phosphorus pollution caused by phosphorus emissions and overuse, this study successfully developed modified anaerobic sludge biochar (ESBC-1) using eggshells as a calcium source, where eggshell-derived CaCO₃ was thermally decomposed into CaO at 700 °C and further hydrolyzed to Ca(OH)₂ in aqueous systems—providing sufficient Ca^2^⁺ for phosphate capture, and determined the optimal preparation conditions to be a mass ratio of 1:1 for eggshell powder to sludge powder and calcination at 700 °C. Under ideal conditions (1 g/L, pH 3–10), with no significant adsorption capacity loss (fluctuation < 8%) even at extreme pH values, the phosphate adsorption capacity of ESBC-1 was 97.74 mg/g, which was 7.9 times higher than that of unmodified biochar. The adsorption process of ESBC-1 complied with the pseudo-second-order kinetic model (R^2^ = 0.9988) —evidencing that chemical adsorption (rather than physical diffusion) dominated the rate-limiting step and the Freundlich isotherm model (R^2^ = 0.9763) —indicating multi-layer adsorption on heterogeneous active sites of ESBC-1. The Langmuir model estimated the maximal adsorption capacity at 298K to be 529.10 mg/g, which was 1.84 times more than that of the unmodified group. The adsorption mechanism mainly involved the Ca^2^ ⁺ produced by the hydrolysis of the material’s surface and phosphate, resulting in the formation of Ca_5_(PO_4_)_3_(OH) via chemical precipitation for phosphorus fixation. This research utilized sludge and eggshells as raw materials to develop a novel phosphorus adsorbent, not only solving the environmental disposal problem of two typical solid wastes but also reducing adsorbent production costs compared with commercial alternatives, offering a new approach for the resource use of these materials and demonstrating promising potential for phosphorus pollution mitigation. Future work could focus on optimizing ESBC-1’s cyclic stability (e.g., via surface coating) to expand its practical application in long-term wastewater treatment.

## Supporting information

S1 Table Adsorption kinetics for the adsorption of phosphate by biochar. (XLSX)

S2 Table Adsorption isotherm for the adsorption of phosphate by biochar. (XLSX)

S3 Table Effect of different mass ratios on the adsorption properties of phosphate. (XLSX)

S4 Table Effect of dosage on phosphate adsorption by ESBC-1. (XLSX)

S5 Table Effect of initial pH on phosphate adsorption by ESBC-1. (XLSX)

S6 Table Effect of coexisting ions on phosphate adsorption by ESBC-1. (XLSX)

S7 Table Phosphate adsorption performance of ESBC-1 and SBC in real biogas slurry. (XLSX)
